# Studies on the Anti-Inflammatory Effect and Its Mechanisms of Sophoridine

**DOI:** 10.1155/2014/502626

**Published:** 2014-04-09

**Authors:** Xiumei Huang, Bo Li, Lianzhong Shen

**Affiliations:** ^1^National Institutes for Food and Drug Control, Beijing 100050, China; ^2^Qinghai Province Institute for Food and Drug Control, Xining 810003, China

## Abstract

This work is to study the anti-inflammatory effect and its mechanisms of sophoridine in vitro and in vivo. For this aim, the influences of sophoridine on several inflammatory mediators were investigated. Excessive inflammatory response in vitro model was developed by using lipopolysaccharide (LPS) to stimulate the mouse peritoneal macrophages and HL-60 cells to produce IL-6 and IL-8. Carrageenin-induced mouse paw edema model was used as inflammatory response in vivo model. MTT method, ultraviolet spectrophotometric method, and radioimmunoassay were used to measure the changes of TNF**α**, IL-6, PGE_2_, and IL-8 in in vitro cell culture supernatant or in the local inflammatory exudates. The results showed that sophoridine inhibited the production of IL-8 in in vitro cell culture supernatant and inhibited the production of TNF**α**, PGE_2_, and IL-8 in the local inflammatory exudates but had no significant effects on the production of IL-6 in vitro and in vivo. It is demonstrated that sophoridine's anti-inflammatory effect was due to its ability to inhibit the production of cytokine and inflammatory mediators.

## 1. Introduction

Inflammation is a complex physiological and pathological process accompanied by the activation of the immune system, local vascular system, and various cells within the damaged tissue. During an inflammatory process cells become activated and several mediators, such as inflammatory cytokines (TNF*α*, IL-6, and IL-8) and inflammation media (PGE_2_, histamine), are released.

The inflammation model of carrageenin-induced edema is usually used to assess the activity of natural products in inhibiting the pathological changes associated with acute inflammation. Carrageenin can induce acute inflammation by increasing infiltration of phagocytes and oxidative stress as well as release of inflammatory mediators.

Sophoridine (SRI, C_15_H_24_N_2_O, relative molecular weight 248.36) is a quinolizidine alkaloid. SRI is also called matridine-15-one (allomatrine). It exists in overground part of Leguminous plant* Sophora alopecuroides *L., overground part of* Euchresta japonica *Benth., and root of* Sophora alopecuroides* Ait. It has been reported that SRI has marked pharmacological activity on anti-inflammatory [1–4], antitumor [5–7], and antivirus [8, 9]. In our preliminary studies [10–13], we had reported some of the anti-inflammatory effects and the mechanism of SRI, including the inhibitory effects on TNF*α* production by murine peritoneal macrophages, the effect on COX in supernatant of cultured macrophages, the effect on leukotrienes (LTC_4_ and LTB_4_) biosynthesis in cells, and the effect on histamine in isolated tracheal smooth muscle and the isolated ileum of guinea pig. This work is to study the anti-inflammatory action and its mechanisms of sophoridine on cells and in vivo models continuously. For this aim, the influences of sophoridine on several inflammatory mediators IL-6 and IL-8 in LPS-stimulated mouse peritoneal macrophages and HL-60 cells and the influences of sophoridine on TNF*α*, IL-6, IL-8, and PGE_2_ in the inflammatory exudates of carrageenin-induced mouse paw edema model were investigated.

## 2. Materials

Sophoridine (SRI, white crystal, freely soluble in water) was provided by Pharmacology Department of National Institutes for Food and Drug Control. Ibuprofen (Batch no. 2000704) was purchased from Anhui Huaen Pharm Co. Ltd). Actinomycin D, lipopolysaccharide (LPS) (Batch no. L2880), thiazolyl blue (MTT), and arachidonic acid (AA) (A3555-10MG) were purchased from Sigma. Brewer thioglycollate medium (BTM) was the product of Difco. RPMI-1640 culture medium was purchased from Gibco. Fetal bovine serum (FBS) was purchased from Hyclone. Carrageenin (Batch no. 021M0038V) was purchased from Sigma. rhlL-6 was provided by Bioassay Department of National Institutes for Food and Drug Control. L929 cell strain (Mouse fibroblast cell strain) and HL-60 cell strain (Human promyelocytic leukemia cells) were provided by Pharmacology Department of National Institutes for Food and Drug Control. 7TD1 cell strain (IL-6 dependent cell strain) was kindly provided as a gift by Academician Beifen Shen (Worked in the third institute of Academy of Military Science). ^125^I-lL-8 radioimmunoassay kit was purchased from East Asia Immune Technology Institute (Beijing).

## 3. Instruments

RS-232C Microplate Reader (Labsystems, Finland), IMT-2 phase-contrast microscope (Olympus, Japan), Radioimmunoassay counter (LKB-Wallac CliniGamma 1272-002, Finland), Low temperature normal speed centrifuge (Hettich, Germeny), MP230 pH meter (METTLER, Switchland).

## 4. Animals

Male C57BL/6 mice (6~8 weeks) and male and female Kunming mice, body weight 18~23 g, were obtained from Experimental Animal breeding Center of National Institutes for Food and Drug Control.

## 5. Methods

### 5.1. The Influences of SRI on the Production of IL-6 in LPS-Stimulated Mouse Peritoneal Macrophages: Preparation of the Mouse Peritoneal Macrophages and Calculation of the SRI Concentration Range Which Does Not Influence the Normal Growth of Peritoneal Macrophages

The methods were the same as previously described [14]. Briefly, each male C57BL/6 mice was given an intraperitoneal injection of 1 mL 3% sodium thioglycollate solution. The animals were sacrificed after 4 days. The peritoneal macrophages were washed out with phosphate buffer solution (PBS) under a sterile environment and were washed twice with RPMI-1640 (contained 5% FBS). The cells were counted and adjusted to 2 × 10^6^ cells per mL.

The 96-cell culture plate, which contained 100 *μ*L macrophage suspension (2 × 10^6^ cells/mL) each well, was incubated at 37 ± 0.5°C under 5% CO_2_ humidified air for 2 hours. 100 *μ*L SRI solutions (concentration range 1 × 10^−8^~1 × 10^−6^ mol/L) were added and the plate was incubated continually for 20 hours under the same condition. 50 *μ*L MTT (2.5 mg/mL) was added each well and the plate was incubated for 4 hours. The supernatant solutions were discarded; 100 *μ*L dimethyl sulfoxide (DMSO) was added each well and mixed thoroughly. The OD values were determined at 570 nm on Microplate Reader after 5 minutes.

### 5.2. The Culture of Mouse Peritoneal Macrophages and Drug Treatment

200 *μ*L macrophage suspension (2 × 10^6^ cells/mL) was added to 48-cell culture plate and incubated for 2 hours at 37 ± 0.5°C in 5% CO_2_ humidified air. The nonadherent cells were washed away with PBS. 200 *μ*L RPMI-1640 culture medium (contained 10% FBS) was added to each well, and 200 *μ*L SRI solutions at different concentrations were added. After 0.5 hours of incubation, 50 *μ*L irritant agent lipopolysaccharide (LPS, 25 *μ*g/mL) was added to each well. Incubate the plate again for 24 hours before the supernatants were collected for the measurement of IL-6.

### 5.3. Determination of IL-6 [15]

50 *μ*L supernatant was added to 96-cell culture plate, and then 50 *μ*L 7TD1 cell suspension (1.2 × 10^5^ cells/mL) was added. After 48 hours incubation, MTT (5 mg/mL) 20 *μ*L was added to each well, and incubated for 4 hours. After Centrifuged under low temperature, the supernatant discarded, then DMSO 100 *μ*L was added to each well, mixed and detected the OD values at 570 nm on Microplate Reader. The decrease of the OD value in drug group indicated that the production of IL-6 in mouse peritoneal macrophages is inhibited by the drug, which was expressed in inhibition percentage. Inhibition percentage (%) = (OD_LPS_ − OD_Drug_)/OD_LPS_ × 100%. Samples were assayed in triplicate and results are reported at means (±SD).

### 5.4. The Influence of SRI on the Production of IL-8 in LPS-Stimulated HL-60 Cells: Calculation of the SRI Concentration Range Which Does Not Influence the Normal Growth of HL-60 Cells

The methods were as previously described [14]. Briefly, 100 *μ*L HL-60 cell suspension (10^5^ cells/mL) was added to the 96-cell culture plate and incubated at 37 ± 0.5°C under 5% CO_2_ humidified air for 2 hours. 100 *μ*L SRI solutions (concentration range 1 × 10^−8^~1 × 10^−6^ mol/L) were added; the plate was incubated continually for 20 hours under the same condition. 50 *μ*L MTT (2.5 mg/mL) was added each well; the plate was incubated for 24 hours. The supernatant solutions were discarded after centrifuging under low temperature. 100 *μ*L dimethyl sulfoxide (DMSO) was added each well and mixed thoroughly; the OD values were determined at 570 nm on Microplate Reader after 5 minutes.

### 5.5. The Culture of HL-60 Cells and Drug Administration

HL-60 cells were cultured in RPMI-1640 culture medium (containing 10% FBS) and were subcultured after one or two days. The HL-60 cells in logarithm stage were washed with D-Hunks solution and counted. The concentration of HL-60 cells was adjusted to 1 × 10^9^ cell/L before use. 200 *μ*L HL-60 cell suspension was added to 48-cell culture plate. 200 *μ*L different concentrations of SRI were added and incubated for 20 minutes at 37 ± 0.5°C under 5% CO_2_ humidified air. 50 *μ*L irritant agent lipopolysaccharide (LPS, 90 *μ*g/mL) was added, and the reference was added with the same amount of solvent instead of LPS and incubated again for 24 hours before the supernatant was collected under low temperature. The supernatant was stored at −20°C for the measurement of IL-8.

### 5.6. Determination of IL-8

The IL-8 was measured by radio immunity with a ^125^I-lL-8 radioimmunoassay kit. The decrease of the IL-8 amount in drug group indicated that the production of IL-8 in HL-60 cells is inhibited by the drug which was expressed in inhibition percentage, inhibition percentage (%) = (IL-8_LPS_ − IL-8_Drug_)/IL-8_LPS_ × 100%. Samples were assayed in triplicate and results are reported at means (±SD).

### 5.7. The Influences of SRI on Carrageenin-Induced Mouse Paw Edema and the Contents of TNF*α*, IL-6, IL-8, and PGE_2_ in the Local Inflammatory Exudates: Effect in Carrageenin-Induced Mouse Paw Edema Model

Male and female Kunming mice (half and half, body weight 18~23 g) were used and divided into four groups (twenty per group). Two dosages of SRI (LD_50_/5 and LD_50_/20) were given orally for 5 days (one time for each day). LD_50_ of SRI is 243 mg/kg (intragastric administration). Ibuprofen was used as a positive control. 30 minutes after the last intragastric administration, 30 *μ*L of 1% carrageenan solution (solvent: 0.9% saline solution) was injected intradermally into the plantar of right hind paw. Mice were killed after 5 hours; the paws were cut at joint and weighted. The degree of swelling was expressed in the difference between the left paw weight and right paw weight. Inhibition percentage (%) = (swelling  degree_Model_ − swelling  degree_Drug_)/swelling  degree_Model_ × 100%.

### 5.8. Sample Preparation for TNF*α*, IL-6, IL-8, and PGE_2_ Determination

Swollen paws of the mice were weighted, skinned, and soaked into 5 mL physiological saline at 4°C for 1 hour after cut into pieces. The swollen paw pieces were discarded and the soak solution was centrifuged. The supernatant was filtered with 0.22 *μ*m filter to remove bacteria before analysis.

### 5.9. Determination of TNF*α*

The methods were as previously described [10]. Briefly, 100 *μ*L RPMI-1640 culture solution (contained actinomycin D 2 *μ*g/mL) was added to 96-cell culture plate which is covered with a monolayer L929 cell. Then 100 *μ*L RPMI-1640 culture solution or 100 *μ*L supernatant obtained under “*Samples for TNF*α*, IL-6, IL-8, and PGE*_*2*_ *determination*” was added and incubated for 20 hours at 37 ± 0.5°C under 5% CO_2_ humidified air. 50 *μ*L MTT (2.5 mg/mL) was added, incubated for 4 hours. After the supernatant discarded, 100 *μ*L DMSO was added to each well and mixed thoroughly. Then the OD values were read at 570 nm on Microplate Reader in 5 minutes.

The RPMI-1640 culture solution wells were used as the reference. The activity of TNF*α* in samples was expressed in cytotoxic percent. Cytotoxic percent (%) = (1 − OD_sample_/OD_reference_) × 100. The inhibition percentage indicated the degree of depression on the production of TNF*α*. Inhibition percentage (%) = (1 −  cytotoxic percent_sample_/cytotoxic percent_reference_) × 100%.

### 5.10. Determination of IL-6 and IL-8

The detection methods were the same as described under IL-6 and IL-8 determination in mouse peritoneal macrophages and HL-60 cells.

### 5.11. Determination of PGE_2_

The methods were as described in [16]. Briefly, added 2 mL 0.1 mol/L KOH methanol solution to 0.1 mL supernatant obtained under “*Samples for TNF*α*, IL-6, IL-8 and PGE*_*2*_ *determination*”. Then stored at 50°C for 20 minutes until isomerization reaction finished. Added methanol up to the volume of 25 mL. Absorbency value of the mixture, which represented the content of PGE_2_, was determined at 278 nm on Microplate Reader.

## 6. Results

### 6.1. Finding of the SRI Concentration Range Which Does Not Influence the Normal Growth of Peritoneal Macrophages and HL-60 Cells by MTT Method

The result showed that, in the SRI concentration range of 1 × 10^−8^~1 × 10^−6^ mol/L, there was no significant difference between the absorbance of the experimental groups and reference well which contained RPMI-1640 culture solution. The result indicated that SRI had no discernible effects on the growth of macrophage and HL-60 cells within the experimental concentration range.

### 6.2. The Influences of SRI on the Production of IL-6 and IL-8 in LPS-Stimulated Mouse Peritoneal Macrophages and HL-60 Cells

The results indicated that in the range of 1 × 10^−8^~1 × 10^−6^ mol/L, SRI could inhibit the production of IL-6 and IL-8 in LPS-stimulated mouse peritoneal macrophages and HL-60 cells in a dose-dependent manner. The IL-6 content of each SRI group had no significant difference compared with that of LPS group. But the IL-8 content of each SRI group was significantly lower than that of LPS group (*P* < 0.01). The results were listed in Table 1.

### 6.3. The Influences of SRI on Carrageenin-Induced Mouse Paw Edema Model and the Contents of TNF*α*, IL-6, IL-8, and PGE_2_ in the Local Inflammatory Exudates

Pretreatment with SRI at doses of 48.60 and 12.15 mg/kg body weight markedly attenuated paw edema after carrageenin stimulation. When the dosage is one fifth of LD_50_, SRI could inhibit the production of IL-6 and IL-8 in the local inflammatory exudates (*P* < 0.05), and SRI could significantly inhibit the production of PGE_2_ in the local inflammatory exudates (*P* < 0.01). When the dosage is one twentieth of LD_50_, SRI could inhibit the production of PGE_2_ (*P* < 0.05) but could not inhibit the production of IL-6 and IL-8. The results are listed in Tables 2 and 3.

## 7. Discussion

It has been reported [1] that SRI had some immunoregulation effect on mouse immunologic function. It could enhance the mouse macrophage phagocytic function and antagonize the immunosuppression induced by cyclophosphamide. Our previous work [10–13] had proved that SRI could significantly inhibit the production of TNF*α* and PGE_2_ in LPS-stimulated mouse peritoneal macrophages in a concentration dependent manner. And SRI had little effect on the biosynthesis of LTC_4_ but inhibited the biosynthesis of LTB_4_ obviously. In a certain range of concentration, SRI could significantly inhibit the contract of isolated guinea pigs bronchial smooth and ileum caused by histamine.

Based on these preliminary results, the current work is to study the anti-inflammatory effect and its mechanism of sophoridine on cells, isolated organ, and in vivo models and to discuss the relationship between anti-inflammatory effect in vivo and inflammation media in cells.

The results showed that in a certain concentration range, SRI could significantly inhibit the production of TNF*α*, PGE_2,_ and IL-8 in LPS-stimulated mouse peritoneal macrophages and HL-60 cells, and the inhibition effect was dose dependent. But the inhibition effect on the production of IL-6 was not significant. SRI markedly attenuated paw edema after carrageenin stimulation. Similarly, SRI could significantly inhibit the production of TNF*α*, PGE_2,_ and IL-8 in the local inflammatory exudates in dose-dependent manner but could not inhibit the production of IL-6 in the local inflammatory exudates.

In summary, SRI showed inhibitory effects on inflammatory cytokines (TNF*α*, IL-6, and IL-8) and inflammation media prostaglandin E_2_ (PGE_2_) both in cell culture supernatant (in vitro) and in the local inflammatory exudates (in vivo). Based on the experimental results obtained from both in vitro and in vivo models, the anti-inflammatory effect of SRI might be attributed to its ability to inhibit the production of cytokines (TNF*α*, IL-8, and LTB_4_) and inflammation media (PGE_2_ and histamine) in the initial phase of inflammation.

## Figures and Tables

**Table 1:** The results of SRI inhibited the production of IL-6 and IL-8 in LPS-stimulated mouse peritoneal macrophages and HL-60 cells (*n* = 9).

Group	IL-6		IL-8	
	OD value (mean ± SD)	Inhibition (%)	Content (ng/mL) (mean ± SD)	Inhibition (%)
Blank	1.408 ± 0.050^(2)^		20.2700 ± 1.3110^(2)^	
LPS (5 *μ*g/mL)	1.627 ± 0.013		39.9700 ± 2.6230	
LPS + SRI (1 × 10^−8^)	1.508 ± 0.079	7.3	13.910 ± 2.474^(2)^	65.2
LPS + SRI (1 × 10^−7^)	1.436 ± 0.099	11.7	13.230 ± 1.167^(2)^	66.9
LPS + SRI (1 × 10^−6^)	1.431 ± 0.081^(1)^	12.0	7.787 ± 1.910^(2)^	80.5

Compared with LPS, ^(1)^ *P* < 0.05, ^(2)^ *P* < 0.01.

**Table 2:** The influences of SRI on carrageenin-induced mouse paw edema model (*n* = 20).

Group	Dosage (mg/kg)	Difference between left and right paw (mg) (mean ± SD)	Inhibition (%)
Model		0.083 ± 0.211	
Ibuprofen	176.5	0.039 ± 0.009^(1)^	53.01
SRI (LD_50_/5)	48.60	0.057 ± 0.010^(1)^	31.32
SRI (LD_50_/20)	12.15	0.058 ± 0.019^(1)^	30.12

Compared with model group, ^(1)^ *P* < 0.01.

**Table 3:** The influences of SRI on the production of TNF*α*, IL-6, IL-8, and PGE_2_ in the local inflammatory exudates of carrageenin-induced mouse paw edema model (*n* = 12).

Group	Dosage (mg/kg)	TNF (OD value) (mean ± SD)	IL-6 (OD value) (mean ± SD)	PGE_2_ (OD value) (mean ± SD)	IL-8 (ng/mL) (mean ± SD)	Inhibition (%)			
						TNF*α*	IL-6	PGE_2_	IL-8
Control		0.680 ± 0.011	1.341 ± 1.156						
Model		0.532 ± 0.023	3.379 ± 0.261	0.303 ± 0.008	0.267 ± 0.081				
Ibuprofen	176.5	0.437 ± 0.025^(2)^	2.413 ± 0.210	0.170 ± 0.029^(2)^	0.146 ± 0.042^(2)^	−64.5	28.6	43.7	45.3
SRI	48.60	0.564 ± 0.030	3.167 ± 0.090^(2)^	0.193 ± 0.011^(2)^	0.210 ± 0.044^(1)^	21.6	6.3	36.4	21.3
SRI	12.15	0.547 ± 0.022	3.132 ± 0.157^(1)^	0.218 ± 0.040^(1)^	0.256 ± 0.073	10.1	7.3	27.9	4.1

Compared with model group, ^(1)^ *P* < 0.05, ^(2)^ *P* < 0.01.
